# Feasibility and outcomes of using DIALOG+ in primary care to improve quality of life and mental distress of patients with long-term physical conditions: an exploratory non-controlled study in Bosnia and Herzegovina, Colombia and Uganda

**DOI:** 10.1186/s12875-023-02197-0

**Published:** 2023-11-16

**Authors:** Francois van Loggerenberg, Dickens Akena, Racheal Alinaitwe, Harriet Birabwa-Oketcho, Camilo Andrés Cabarique Méndez, Carlos Gómez-Restrepo, Alma Džubur Kulenović, Nejra Selak, Meliha Kiseljaković, Seggane Musisi, Noeline Nakasujja, Nelson K. Sewankambo, Stefan Priebe

**Affiliations:** 1 Youth Resilience Unit, Wolfson Institute of Population Health, Queen Mary University of London, London, UK; 2 Department of Psychiatry, Makerere University College of Health Sciences, Kampala, Uganda; 3 Butabika National Referral Mental Hospital, Kampala, Uganda; 4 Department of Clinical Epidemiology and Biostatistics, Pontificia Universidad Javeriana, Bogotá, Colombia; 5 Departments of Clinical Epidemiology and Biostatistics and Psychiatry and Mental Health, Pontificia Universidad Javeriana, Bogotá, Colombia; 6 Clinical Center University of Sarajevo, Sarajevo, Bosnia and Herzegovina; 7 Primary Care Center Zenica, Zenica, Bosnia and Herzegovina; 8 Emergency Medical Center of Canton Sarajevo, Sarajevo, Bosnia and Herzegovina; 9 Department of Internal Medicine, Makerere University College of Health Sciences, Kampala, Uganda; 10 Unit for Social and Community Psychiatry, East London NHS Foundation Trust, London, UK

**Keywords:** Global mental health, Primary care, Psychosocial interventions, Resource-oriented approach, LMICs, Solution-focused, Quality of life, DIALOG+

## Abstract

**Introduction:**

The management of long-term physical conditions is a challenge worldwide, absorbing a majority resources despite the importance of acute care. The management of these conditions is done largely in primary care and so interventions to improve primary care could have an enormous impact. However, very little data exist on how to do this. Mental distress is frequently comorbid with long term physical conditions, and can impact on health behaviour and adherence, leading to poorer outcomes. DIALOG+ is a low-cost, patient-centred and solution-focused intervention, which is used in routine patient-clinician meetings and has been shown to improve outcomes in mental health care. The question arises as to whether it could also be used in primary care to improve the quality of life and mental health of patients with long-term physical conditions. This is particularly important for low- and middle-income countries with limited health care resources.

**Methods:**

An exploratory non-controlled multi-site trial was conducted in Bosnia and Herzegovina, Colombia, and Uganda. Feasibility was determined by recruitment, retention, and session completion. Patient outcomes (quality of life, anxiety and depression symptoms, objective social situation) were assessed at baseline and after three approximately monthly DIALOG+ sessions.

**Results:**

A total of 117 patients were enrolled in the study, 25 in Bosnia and Herzegovina, 32 in Colombia, and 60 in Uganda. In each country, more than 75% of anticipated participants were recruited, with retention rates over 90% and completion of the intervention exceeding 92%. Patients had significantly higher quality of life and fewer anxiety and depression symptoms at post-intervention follow-up, with moderate to large effect sizes. There were no significant improvements in objective social situation.

**Conclusion:**

The findings from this exploratory trial suggest that DIALOG+ is feasible in primary care settings for patients with long-term physical conditions and may substantially improve patient outcomes. Future research may test implementation and effectiveness of DIALOG+ in randomized controlled trials in wider primary care settings in low- and middle-income countries.

**Trial registration:**

All studies were registered prospectively within the ISRCTN Registry. ISRCTN17003451, 02/12/2020 (Bosnia and Herzegovina), ISRCTN14018729, 01/12/2020 (Colombia) and ISRCTN50335796, 02/12/2020 (Uganda).

## Introduction

The management of long-term physical conditions is a challenge worldwide, utilising the majority of available resources despite the importance of acute care. In the United Kingdom (UK), the National Health Service estimates that the treatment and care of patients with long-term physical conditions absorbs as much as 70% of the resources allocated to acute and primary care [1]. The management of these conditions is done largely in primary care and so interventions to improve primary care could have an enormous impact. However, very little data exist on how to improve the management of these conditions in primary care.

Psychological distress and poor quality of life has been associated with a range of long-term physical conditions like cardiovascular disease, HIV/AIDS, cancer and diabetes [2–5]. Mental distress is frequently co-morbid with these long-term physical conditions [6–11]. Mental and physical disorders share a number of risk factors, they can bi-directionally cause or facilitate each other [12], and mental distress can impact on health behaviour and treatment adherence and therefore worsen physical problems. Interventions addressing mental distress in patients with long-term physical illnesses could potentially improve their quality of life and overall functioning [13]. This calls for the integration of psychosocial interventions in primary care of patients with long-term physical conditions. Such integration has been considered a priority in the overall provision of mental health care [14], and various efforts have been made towards achieving it [15]. However, a number of challenges remain. They include insufficient funding, a shortage of appropriately qualified staff, and a high demand for services leaving little time for additional activities and interventions. Another core challenge is a lack of evidence-based psycho-social interventions that are feasible and effective in primary care settings, especially within the context of the health care systems in low- and middle-income countries (LMICs) [16–20].

To improve outcomes in primary settings, a low-cost approach is required that can be applied in routine settings and does not require setting up new specialised services. One approach that has been specifically developed to make routine patient-clinician meetings more effective, is DIALOG+. Although originally designed, studied and evidence-based in secondary mental health care services, its generic nature suggests that it may also be beneficial for patients with various chronic conditions in primary care.

## The DIALOG+ intervention

Rather than focussing on addressing patient deficits as many psychosocial interventions do, some focus rather on the strengths of the patients and on mobilising their existing personal and social resources to address their difficulties. These approaches have been labelled resource-oriented [21], and DIALOG+ is one such intervention. The content and development of the DIALOG+ intervention have been described in detail elsewhere [22]. In brief, DIALOG+ is a resource-oriented and evidence-based intervention, which leverages existing social and personal resources in order to improve the quality of life, originally developed for patients with severe mental illnesses [23]. This is done by structuring and focusing part of the patient-clinician conversation during routine clinical meetings. At the beginning of each session, patients rate their satisfaction with eight life domains (mental health, physical health, job situation, accommodation, leisure activities, family/partner, friendships, and personal safety) and three treatment aspects (medication, practical help, and meetings with professionals) on the tablet using a seven-point visual scale ranging from ‘totally dissatisfied’ to ‘totally satisfied’. An overview of the ratings is displayed and can be compared with any previous rating. This allows patient and clinician to discuss current strengths and problems. Patients then select up to three domains to explore in more detail in the given meeting. Each concern is addressed following a four-step, solution-focused approach: (1) understanding the patient’s concerns and identifying what works well; (2) looking forward and considering best case scenarios as well smallest tangible steps forward; (3) exploring actions that the patient, clinician, and others can take; and (4) agreeing on actions.

## Aims and objectives

The primary aim of this study was to explore the feasibility and outcomes of DIALOG+ in primary care settings in LMICs with patients with long-term physical conditions and poor quality of life. This would be both to support implementation of DIALOG+ in these settings, and to inform the development of future fully powered randomised controlled trials in those settings where more data would be beneficial to supporting roll out.

## Methods

The details of the study development, protocol, design and background have been published elsewhere [22]. An exploratory non-controlled multi-site study was conducted in each of Bosnia and Herzegovina, Colombia and Uganda, with a target enrolment of at least 30 patients in each country.

The NIHR-funded Global Health Group on developing psycho-social interventions in low- and middle-income countries was established in 2017 to explore the application of resource-oriented interventions to improve community mental health care in people with severe mental illness [24]. The group was originally a collaboration between Queen Mary University of London in the UK and three original partner countries – Bosnia and Herzegovina, Colombia, and Uganda. The group was later expanded to include additional studies in Argentina, Peru and Pakistan. This group was specifically testing three resource-oriented interventions: Multi-family groups, Volunteer befriending, and DIALOG+. During this work, the investigators identified and discussed the unmet need for psychosocial support in order to improve primary care delivery to support good mental and physical health. Of the three interventions being assessed, DIALOG+ was identified as being the most likely to be implemented within primary care as it leverages the routine meetings that were already happening and this intervention was specifically designed to make routine care meetings therapeutically effective, but this had not been tested in primary care settings.

Clinicians from the clinics who volunteered to take part in the study received training and manuals describing the details of the intervention [25]. They were provided with a small incentive for participation, as reviewed and approved by the local ethics committee. One of the strengths of DIALOG+ is how simple it is use, and how efficiently it can be trained on. In the UK, the standard method of training is that clinicians receive a single training session lasting up to 2 h which involves learning about the developmental background of the approach, the evidence base which underpins it, and testimony from service users and carers about its benefits. The session ends with around 30 min of skills practice where clinicians practice the application of the intervention using clinical vignettes and role play. Much of this training is delivered online. For this trial, the team made use of the experience and capacity of peers who had been trained in the main NIHR Group Health Group studies in country [26]. Clinicians received a single training session, delivered face-to-face by team members experienced in using DIALOG+, and including role playing as done in the UK training. For all settings, clinicians delivered the intervention in the relevant local languages. For Bosnia and Herzegovina this was Bosnian, in Colombia this was Spanish and in Uganda this was Luganda and English. Enrolled participants were offered monthly DIALOG+ sessions at routine clinic visits, over a period of approximately three months. Feasibility was determined by the extent to which the intervention was implemented as planned, and the how well the study recruited and retained participants over the study period. For assessing outcomes, key outcome criteria were assessed at baseline and post-intervention and compared.

The study used a consistent core protocol which ensured comparable implementation across the three country sites. The protocol also provided some flexibility to ensure suitability to the local contexts. This resulted in small differences in the inclusion criteria of patients.

### Patients and procedures

Patients with long-term illnesses such as hypertension, diabetes, cardiovascular disease and chronic obstructive pulmonary disease were targeted for inclusion in the study, as these are often associated with mental distress and can impact on the patient’s quality of life [27].

The following inclusion criteria were used:

1. Adult patients (16–65 in Bosnia and Herzegovina and Colombia; 18 and above in Uganda).
2. At least one long-term physical condition.
3. Poor quality of life (< 5 on the Manchester Short Assessment of Quality of Life (MANSA), < 5.5 in Colombia where previous work with patients indicated that they score slightly higher on the MANSA [26]).
4. Capacity to provide informed consent.
5. Command of the local language.
6. Living within 20 km of the clinic.
7. Having attended the primary care clinic for at least six months.

Given the exploratory and pragmatic nature of the study with the aim of including as many eligible primary care patients as possible, being unable or unwilling to provide informed consent was the only exclusion criterion.

The clinicians were required to be a qualified health professional working in the relevant clinic with no plans to leave their post within four months of recruitment, with no other exclusion criterion.

### Participants and consent

The aim was to recruit at least 30 participants in each country, in line with central limit theorem which states at 30 is the minimum number of participants required to determine meaningful parameter estimates for exploratory studies [28]. Clinicians at the clinic were asked if they would like to take part and those willing were recruited. For the patients, those meeting clinical eligibility while the staff were conducting recruitment activities were approached for screening and consent procedures. All patients and clinicians provided written informed consent prior to any data collection. The process for documenting and the procedures for conducting informed consent, including of patients with low literacy, were reviewed and approved by the local ethics committee. Capacity to consent was assessed at screening. The University of California Brief Assessment of Capacity to Consent (UBACC [29]) was used in Uganda, where it had been previously used. An adapted capacity to consent checklist based on the one published by the British Psychological Society was used in Bosnia and Herzegovina and Colombia for those participants for whom it was felt might be lacking capacity to consent [30]. Patients were not reimbursed for attendance at routine meetings where DIALOG+ was used but were reimbursed for travelling to research interviews and for the time they spent in these interviews which were for data collection only, in line with amounts reviewed and approved by the ethics committees in each country. Although it could not be determined definitively, the fact that the data collection visits were separate from the data collection visits (which only happened at the start and after the end of the intervention) reduces the possible impact of this compensation on participation at their routine meetings. Participation at routine meetings was necessary for clinical care.

### Time periods

After enrolment, the patients received DIALOG+ at their routine clinic appointments, approximately monthly. These sessions were delivered by their healthcare worker, using the DIALOG+ application on a tablet computer. The intervention period was approximately three months (with some flexibility around COVID-19 restrictions which delayed some clinic appointments), and patients received up to three DIALOG+ sessions.

Socio-demographic information, including clinical characteristics for the patients, was collected at baseline. Outcomes were measured at baseline and after the intervention period.

### COVID adjustments

At the time of planning for this study, it was not clear what the impact of ongoing and potentially changing pandemic restrictions would be [22]. It was anticipated, for example, that initially all sessions in Colombia would have been delivered remotely. As it happened, it was only necessary to do this for the three-month data follow-up sessions. In Bosnia and Herzegovina, recruitment of patients was delayed due to movement restrictions during partial lockdowns. In addition, recruited clinicians had increased workloads at certain times due to pandemics, so that some intervals between routine meetings were longer than the planned one-month period. There was no impact on activities due to COVID in Uganda.

### Measures

The feasibility of the intervention was assessed as patient and clinician recruitment and attrition (with reasons for refusal and loss to follow-up) as well as the frequency and duration of sessions.

Three core feasibility criteria were pre-defined:

1. at least 75% of the anticipated 30 participants per country recruited, and,
2. at least 75% of approached and eligible participants enrolled in the study, and,
3. At least 75% retention in the study.

Completion of at least two thirds of the planned sessions was included as an additional feasibility criterion.

Socio-demographic information was captured on a questionnaire.

Outcome measures were:

1. Subjective quality of life was rated on the Manchester Short Assessment of Quality of Life (MANSA) [31].
2. Symptoms of depression were assessed on the Patient Health Questionnaire (PHQ-8), a brief scale with good sensitivity and specificity based on the DSM-IV diagnostic criteria for depressive disorders [32].
3. Symptoms of anxiety were rated on the Generalised Anxiety Disorder Assessment (GAD-7) which has been used in primary care settings showing good validity and reliability [33].
4. The objective social situation of patients was assessed using the Objective Social Outcomes Index (SIX) [34].

These measures were selected to be consistent with the other studies being conducted in the NIHR Group [26, 35, 36], because they have been used in other studies in the settings or local language versions have been validated for use [37–45].

### Data Analysis

Quantitative data were analysed using IBM SPSS Statistics for Windows, Version 28.0.0.0 (190). For age, years old and range was reported. For socio-demographic and clinical characteristics, frequency counts and percentages were prepared. For quantitative outcomes, scores were calculated at baseline and for follow-up post intervention, and repeated measures t-tests were conducted with a p value of < 0.05 to assess changes in the outcomes. Effect sizes were calculated for all outcomes using Cohen’s effect size.

### Settings

The study was conducted in sites in three partner countries, with different arrangement and management of primary care settings, as outlined in greater detail elsewhere [22]. Briefly, these settings were chosen as they formed part of Global Health Group on developing psycho-social interventions in low- and middle-income countries, as described in more detail in the Methods section. They represent three very different but resource-constrained contexts, to assess the generatability of the findings globally. The mental health measures in this study were not available to the treating clinicians and could not play any role in referrals for additional mental health care. No instances of patients being referred for mental health services was recorded in the study.

### Bosnia and Herzegovina

The site in Bosnia and Herzegovina was in the Federation of Bosnia and Herzegovina, where health care provision is delivered through ten cantons each with their own health ministry with the Federal ministry of health providing a guidance role [46, 47]. Within this system, there is a focus on continuity of care. Primary health care services include primary mental health care in the Community Mental Health Centres and family medicine. Outpatient clinics are situated in local communities in order to make sure that primary health care is accessible and available to all citizens. Patients were recruited from the Public Institution Health Care Centre of Sarajevo. The Public Institution of Health Care is the largest institution in the country offering primary health care [48]. The offices of the primary health care centre were used for delivery of the intervention, patient assessments, interviews and focus groups as well as training as they provided confidentiality and privacy. The clinic load in this setting involves each clinician optimally seeing at least 30 patients per day, but this is often as high as 80 due to limited staffing. COVID impacted on clinic numbers and the ability to see patients face-to-face, and this is further described in the ‘COVID adjustments’ section.

### Colombia

The study in Colombia was conducted in Bogotá with patients and clinicians recruited from the Javesalud Institución Prestadora de Servicios de Salud. Initially the plan was to recruit from both their Santa Beatriz and Toberin clinics, but the Toberin clinic shifted to treating only COVID patients and so all participants were recruited from Santa Beatriz. The Colombian healthcare system model places an emphasis on the role of primary care within the national public health strategy. Within the plans in place, the first route to access is provided through primary care, which aims to ensure timely access to medical care. Comorbidity is also managed through the primary care system as there is a shortage of specialists [49]. Another function is to identify the specific care required according to 16 different health risk groups, which includes mental health, violence-related and drug abuse related healthcare issues. However, there is limited capacity in primary care to manage long-term conditions in an integrated way, and expanding this capacity remains a priority. The Santa Beatriz clinic has 6 doctors for outpatient consultation who care for between 108 and 144 patients per day. There was no change to this clinic load over the course of the this study.

### Uganda

In Uganda primary care is delivered by both public and private sectors, with the public sector accounting for around 60% of care delivered [50]. National referral hospitals are the final points of the health services, but the specific referral pathway is often ignored due to a lack of clear gatekeeper control [51]. In practice this means that communities living near the referral hospitals use these as primary health care services, despite the established referral pathways. The study in Uganda was conducted in the outpatient clinics of Mityana district hospital and Masaka regional referral hospital. These outpatient clinics are for long-term physical illnesses, and are run by doctors, clinical officers and senior nurses. In these clinics, the usual load would be 3–5 clinicians working each with around 80–120 patients per clinic day. There weas no change in clinic load over the course of this study.

## Results

### Sample

A total of 117 patients were recruited, 25 in Bosnia and Herzegovina, 32 in Colombia, and 60 in Uganda. The socio-demographic characteristics are summarised in Table 1.

**Table 1:** Demographic data by country

	Bosnia and Herzegovina n = 25	Colombia N = 32	Uganda N = 60
Mean age in years (range)	51 (24–66)	54 (23–64)	57 (19–82)
Sex – Female	17 (68%)	28 (88%)	38 (63%)
Marital Status	9 (36%) Married 8 (32%) Single 4 (16%) Widow(er) 3 (12%) Divorced 1 (4%) Separated	11 (34%) Married 10 (31%) Single 6 (19%) Co-habiting 2 (6%) Separated 2 (6%) Divorced 1 (3%) Widow(er)	29 (48%) Married 11 (18%) Widow(er) 8 (13%) Co-habiting 7 (12%) Single 5 (8%) Separated
Education	1 (4%) Primary 20 (80%) Secondary 4 (16%) Tertiary	5 (16%) Primary 12 (38%) Secondary 6 (19%) Tertiary 9 (28%) Other (not specified)	26 (43%) Primary 20 (33%) Secondary 10 (17%) Tertiary 4 (7%) No formal education
Living situation	20 (80%) Partner or family 4 (16%) Alone 1 (4%) Friends or relatives	18 (56%) Partner or family 11 (34%) Friends or relatives 3 (9%) Alone	54 (90%) Partner or family 3 (5%) Friends or relatives 3 (5%) Alone
Employment	8 (32%) Unemployed 7 (28%) Paid Employment 6 (24%) Retired (due to disability) 2 (8%) Retired (due to age) 1 (4%) Student 1 (4%) Grey economy	14 (44%) Housework 8 (25%) Paid Employed 4 (13%) Retired (due to age) 3 (9%) Unemployed 3 (9%) Paid Employment (Part time)	33 (55%) Paid Employment (Full time) 14 (23%) Unemployed 10 (17%) Paid Employment (Part time) 3 (5%) Retired (due to age)

### Long-term physical condition

The clinical conditions named on the data forms were specifically selected per country to be appropriate to the local context, clinic, and illness mix, and so there is some overlap in the conditions noted. However, the design was intended to recruit from different clinical populations to determine the flexibility of DIALOG+ across a range of clinical conditions. The primary long-term conditions diagnosed in patients, along with secondary or other conditions are noted in Table 2.

**Table 2:** Long-term physical conditions of participants by country

Bosnia and Herzegovina n = 25 n (%)	Colombia n = 32 n (%)	Uganda n = 60 n (%)
10 (40%) Cardiovascular 8 (32%) Osteoporosis/bone density/arthritis 3 (12%) Hyperlipidaemia 2 (8%) Cancer 2 (8%) Impaired lung function 1 (4%) Dental 1 (4%) Obesity 20 (80%) Other: • 2 (10%) hypo-thyroidism • 2 (10%) war injury • 2 (10%) migraine • 2 (10%) gastro-oesophageal reflux	21 (66%) High blood pressure 14 (44%) Diabetes mellitus 1 (3%) Osteoporosis/ arthritis 1 (3%) Hyperlipidaemia 1 (3%) Obesity 7 (22%) Other: • 2 (29%) Hypo-thyroidism • 1 (14%) Osteopenia • 1 (14%) Spondylosis	49 (82%) High blood pressure 43 (72%) Diabetes mellitus 3 (5%) Cardiovascular 1 (2%) Cancer 7 (12%) Other: • 3 (43%) HIV/AIDS • 1 (14%) Allergy • 1 (14%) Hepatitis B • 1 (14%) Hernia • 1 (14%) Ulcers

There are many commonalities across the three clinical settings in terms of socio-demographics and the long-term physical conditions of the participants. The samples are of similar age, and women make up the majority of participants in all three countries. Most of the participants were married and just less than a fifth had tertiary education. A large proportion of the participants lived with their partner or family. There were also some differences, notably in the percentage of participants that are female in Colombia and the proportion who only completed primary school. In terms of long-term physical conditions there was a dominance of cardiovascular and blood pressure given as the primary long-term condition, although this was lower in Bosnia and Herzegovina. Diabetes was also common in Colombia and Uganda. There are some country-specific differences. For example, 10% of participants noting a secondary condition in Bosnia and Herzegovina reported war injuries, whereas 43% of those reporting other conditions in Uganda noted HIV/AIDS as a secondary condition.

### Recruitment and retention

**Fig. 1:**
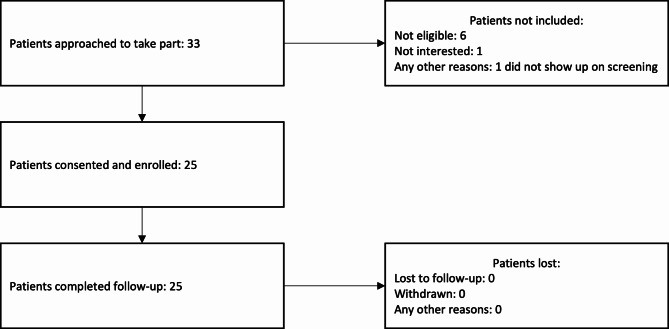
Screening and Retention - Bosnia and Herzegovina

Our feasibility criteria for this study included at least 75% of the anticipated 30 participants per country and at least 75% of the eligible participants enrolled, with at least 75% retention [22]. In Bosnia and Herzegovina 33 participants were approached to take part (Fig. 1). Six were not eligible by MANSA, 1 was not interested, and 1 did not arrive for screening which means that 25 out of 27 eligible (93%) and 25 out of the expected 30 (83%) were recruited, meeting both recruitment criteria. All 25 provided data for the analysis, meaning that retention across the study period was 100%.

**Fig. 2:**
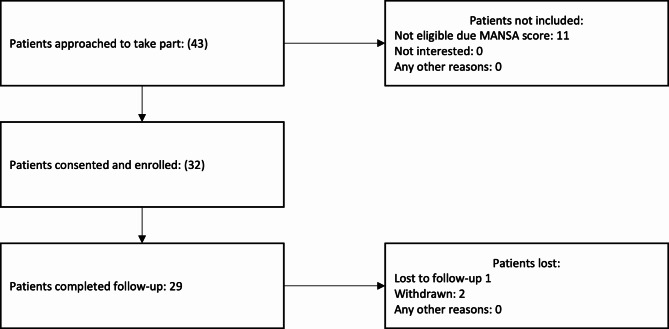
Screening and Retention - Colombia

In Colombia, 43 participants were approached to take part (Fig. 2). Eleven were not eligible as per MANSA scores and 32 were consented and enrolled. This is a recruitment of 100% of the eligible participants, and a slight over recruitment in terms of the anticipated recruitment of 30 participants (107%). Of these, 1 was lost to follow up and 2 withdrew from the study, leaving 29 (91%) retained in the analysis.

**Fig. 3:**
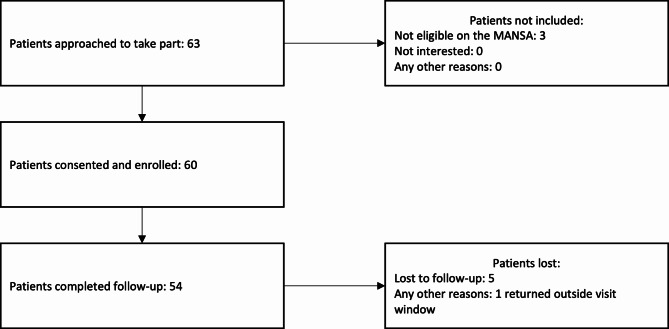
Screening and Retention - Uganda

In Uganda, 63 participants from two clinics were approached to take part in the study, 3 of whom were not eligible as per the MANSA scores leaving 60 to be enrolled, which represents 200% of the anticipated recruitment as well as 100% of those eligible agreeing to take part (Fig. 3). Five participants were lost to follow-up, and one returned outside of the visit window for the follow-up assessment, leaving 54 (90%) in the analysis.

Overall, 108 of 117 (92%) of enrolled participants were retained in the analysis. Thus, the study met both the recruitment core feasibility criteria as well as the retention criterion in each participating country and in the pooled analysis.

### Session completeness

The duration of sessions varied. From as short at 13 to as long as 45 min. The first session was often longer, and this decreased with familiarity with the process. A final feasibility criterion was set to an average of at least two out of three sessions completed as planned. In Colombia, one participant only had one session, one had two sessions and the remaining 28 (88%) had all three sessions. In Bosnia and Herzegovina, only one participant had one session and the remaining 24 (96%) all had all three sessions. In Uganda, 60 participants had one session, 69 had two and 55 (92%) had all three. This means that the study met this additional feasibility criterion.

### Outcomes

Three core primary outcomes are reported in this analysis (Table 3): subjective quality of life (MANSA), objective social situation (SIX), symptoms of anxiety (GAD-7), and symptoms of depression (PHQ-8).

**Table 3:** Patient outcomes

Outcome	Baseline Mean (SD)	Post-intervention Mean (SD)	Cohen’s Effect Size (95%CI)	t-statistic	2-sided p
**MANSA** (N = 108)	3.98 (0.79)	5.16 (0.79)	-1.36 (-1.62, -1.10)	-14.2	**< 0.001**
**GAD-7** (N = 107)	8.98 (6.55)	6.21 (6.04)	0.52 (0.31, 0.73)	5.4	**< 0.001**
**PHQ-8** (N = 108)	9.89 (7.52)	6.89 (6.48)	0.53 (0.33, 0.73)	5.5	**< 0.001**
**SIX** (N = 108)	4.77 (1.21)	4.94 (1.19)	-0.16 (-0.35, 0.03)	-1.7	0.098

Subjective quality of life and symptoms of anxiety and depression improved significantly between baseline and the end of the intervention. The effect size of quality of life changes was large, whilst effect sizes for symptom change were medium. There was also a positive change in the objective social situation which however reflected only a statistical trend and did not reach a significance level of 5%.

## Discussion

This is the first study to examine the feasibility and outcomes of DIALOG+ for patients with long-term physical conditions in primary care settings. The intervention was found to be highly feasible in three participating sites across different countries, meeting all criteria of recruitment, retention and completeness. Patients showed statistically significant and clinically meaningful improvements in subjective quality of life and anxiety and depression symptoms, whilst improvements in the objective social situation reflected only a statistical trend and not a statistical significance. This finding gives hope to the potential impact of a simple and low-cost psychosocial intervention for this very diverse patient population when integrated in primary care and without recourse to separate and resource-intensive specialised services. Instead, it leverages the existing patient-clinician relationship and incorporates a structured, patient-centred and solution-focused approach to communication associated with improvements in quality of life and mental health. These quantitative data will be supplemented by analysis of qualitative data, which is being conducted separately in the partner countries. The qualitative data will provide additional contextual information about how the intervention was experienced by the providers and patients and will also provide more information on the acceptability of the intervention in the specific partner settings.

### Lessons for future research

DIALOG+ can feasibly be delivered by health care providers in the primary care setting, which is reassuring for future fully powered trials. The effect sizes, interpreted cautiously, still provide support to be optimistic about the potential impact of DIALOG+ in diverse settings. Although the intervention can be rolled out with minimal impact on local resources, some training is required, tablets do need to purchased (although the requirements are minimal, and these can be of the cheapest, lowest specification tablets) and a quiet room to deliver the intervention is still required. The latter may be an issue at busier LMIC clinics, and this would also need to be established for a successful fully powered trial to be conducted. It is also recommended that the clinicians receive top-up training if a future trial were to be conducted over a longer follow up period, to encourage reflection and discuss any barriers to implementation that may arise. This additional time and training might be a challenge to adopting DIALOG+ in different settings.

### Strengths and limitations

The study used the same protocol across the three sites on three different continents, and the protocol was well implemented with high recruitment and retention rates. DIALOG+ was used in a pragmatic manner in routine clinical meetings and with heterogeneous samples, and despite this heterogeneity was still associated with good effect sizes. This means that the results should be transferable to real-world settings in different geographical regions, to countries with varying health care systems, and to patients with different characteristics and physical conditions. The clinicians were primary care physicians, who were successfully trained in DIALOG+ in a short period of time and applied the intervention in their regular sessions with the patients. Patients consistently showed improvements with medium to large effect sizes, suggesting that the intervention may be beneficial for this patient group in different world regions and in a range of cultural settings.

The study also has some limitations. Firstly, there was no control group. With a randomized control group receiving treatment as usual, the observed improvements could have been distinguished from potential spontaneous improvements. With an active control group – as it was used in some DIALOG+ trials in mental health care – one could also have controlled for non-specific effects, e.g. of repeated ratings and additional attention. Secondly, the study did not have a follow-up beyond the intervention period to explore the longer-term sustainability of the gains. Finally, the quantitative results do need to be interpreted cautiously as the study was not designed or powered to support inferential analysis. Effect sizes from pilot studies tend to be optimistic and can be unstable due to small sample sizes and this needs to be considered when using these estimates to design fully powered randomized controlled trials [52].

Although this study was conducted in such a way as to match the real-life conditions of the clinics, some differences are inevitable in the context of research. No extra staff were used in this study for the delivery of the intervention, although in most cases a private room was required to deliver the sessions. Training was provided by in-country expertise which would not exist in all contexts, but this could also be delivered remotely as it is in the UK. Computer tablets are not usually provided in these settings and had to be provided for the study. However, DIALOG+ is not resource intensive, and the tablets can be of the least expensive available. Secure storage for the tablets would also have to be available.

### Comparison against the literature

The application of DIALOG+ in this setting is innovative and there are no studies with a very similar method. Thus, it is only possible to compare only against other psycho-social interventions which tend to be very different in their approach. A review of mindfulness-based interventions in primary care found a positive impact on mental health and quality of life [53]. Other studies in looking at integrating psychosocial support for mental health improvement in primary care settings have tended to focus on common mental disease and look at creating capacity within primary care providers to identify, diagnose and refer patients through an established referral pathway [15]. No work has been on resource-oriented psychosocial interventions designed to make the existing clinical care meetings therapeutically effective themselves to improve quality of life with an assessment of the consequent improvement in mental health and wellbeing. Where studies have been done, interventions have usually been illness specific [54–58], whole system or comprehensive, complex and resource intensive [59, 60], and most often look at improvements in quality of life as a secondary outcome of improving aspects of clinical care [61, 62]. As this was the first study testing DIALOG+ in primary care settings, there are no results in the literature that the findings can be directly compared against. However, one can consider the findings in the light of DIALOG+ trials with patients with mental disorders receiving treatment in secondary mental health services [32–34]. As compared against those studies, the effect sizes in this study are substantial, although the intervention period here was only three months and not six months as in most studies in mental health care. Whilst in the absence of a control group the effect size in this study needs to be interpreted with caution, it may be worth noticing that even as compared to the changes in the intervention groups in controlled trials in mental health in high-income countries alone, the effect sizes in this study tend be as large or even larger, particular the one in quality of life.

One can only speculate as to why such a brief and inexpensive intervention may be so beneficial. The intervention might have a particularly strong impact in settings where the provision of routine care is very limited, or the patient-centred and holistic approach of DIALOG+ might contrast with usual patient-clinician communication even more than in the settings of previous mental health studies.

## Conclusions

DIALOG+ is flexible and low cost, requires only brief training and appears feasible with patients with chronic physical conditions in different primary care settings in LMICs. The findings of this study also suggest that its use might lead to clinically relevant improvements in quality of life and symptoms of depression and anxiety in these patient groups. Thus, it might be a suitable and effective approach to be used in primary care, especially in health care settings with limited resources.

Future studies may explore effectiveness and implementation methods of DIALOG+ in primary care in LMICs in controlled trials and with larger samples. Such studies may use DIALOG+ more flexibly and over different time periods than in this study and identify critical components of the intervention that can be strengthened to make it more effective in different contexts.

## Data Availability

Anonymised data arising from the study will be available to external researchers upon reasonable request from the UK Principal Investigator (Professor Stefan Priebe, stefan.priebe2@nhs.net) based on a signed data sharing agreement and only after the publication of the findings of the study by the research team. Study protocols are available on request, and a study protocol has been published [21].

## Abbreviations

GAD-7: Generalised Anxiety Disorder Assessment
HIV/AIDS: Human immunodeficiency virus/acquired immune deficiency syndrome
LMICs: Low- and middle-income countries
MANSA: Manchester Short Assessment of Quality of Life
NIHR: National Institute for Health and Care Research
PHQ-8: Patient Health Questionnaire
SIX: Objective Social Outcomes Index
SPSS: Statistical Package for the Social Sciences
UBACC: University of California Brief Assessment of Capacity to Consent
UK: United Kingdom

## References

[1] NHS. House of Care – a framework for long term condition care. https://www.england.nhs.uk/ourwork/clinical-policy/ltc/house-of-care/.

[2] Barnett K (2012). Epidemiology of multimorbidity and implications for health care, research, and medical education: a cross-sectional study. The Lancet.

[3] Moussavi S (2007). Depression, chronic Diseases, and decrements in health: results from the World health surveys. Lancet.

[4] Fortin M (2004). Multimorbidity and quality of life in primary care: a systematic review. Health and Quality of Life Outcomes vol.

[5] Bayliss EA, Ellis JL, Steiner JF (2005). Subjective assessments of comorbidity correlate with quality of life health outcomes: initial validation of a comorbidity assessment instrument. Health Qual Life Outcomes.

[6] Prince M et al. No health without mental health. Lancet vol. 370 859–877 Preprint at 10.1016/S0140-6736(07)61238-0 (2007). 10.1016/S0140-6736(07)61238-0 17804063

[7] Cassileth BR (1984). Psychosocial Status in Chronic Illness. N Engl J Med.

[8] Strine TW, Chapman DP, Kobau R, Balluz L, Mokdad AH (2004). Depression, anxiety, and physical impairments and quality of life in the U.S. noninstitutionalized population. Psychiatric Serv.

[9] Ngo VK et al. Grand challenges: integrating Mental Health Care into the non-communicable Disease Agenda. PLoS Med 10, (2013). 10.1371/journal.pmed.1001443 PMC3653779 23690753

[10] Kagee A, Martin L (2010). Symptoms of depression and anxiety among a sample of South African patients living with HIV. AIDS Care - Psychological and Socio-Medical Aspects of AIDS/HIV.

[11] Andersen L, Kagee A, O’Cleirigh C, Safren S, Joska J (2015). Understanding the experience and manifestation of depression in people living with HIV/AIDS in South Africa. AIDS Care - Psychological and Socio-Medical Aspects of AIDS/HIV.

[12] Patel V (2009). Integrating mental health care with chronic Diseases in low-resource settings. Int J Public Health.

[13] Noël PH (2004). Depression and comorbid Illness in elderly primary care patients: impact on multiple domains of health status and well-being. Ann Fam Med.

[14] Patel V, Saxena S (2019). Achieving universal health coverage for mental disorders. The BMJ vol.

[15] Petersen I et al. Evaluation of a collaborative care model for integrated primary care of common mental disorders comorbid with chronic conditions in South Africa. BMC Psychiatry 19, (2019). 10.1186/s12888-019-2081-z PMC6448306 30943947

[16] Petersen I, Ssebunnya J, Bhana A, Baillie K (2011). Lessons from case studies of integrating mental health into primary health care in South Africa and Uganda. Int J Ment Health Syst.

[17] Jack H (2014). Closing the mental health treatment gap in South Africa: a review of costs and cost-effectiveness. Glob Health Action.

[18] Lund C, Petersen I, Kleintjes S, Bhana A. Mental health services in South Africa: Taking stock. African Journal of Psychiatry (South Africa) vol. 15 402–405 Preprint at 10.4314/ajpsy.v15i6.48 (2012). 10.4314/ajpsy.v15i6.48 23160613

[19] Petersen I (2017). Strengthening mental health system governance in six low- and middle-income countries in Africa and South Asia: challenges, needs and potential strategies. Health Policy Plan.

[20] Docrat S, Besada D, Cleary S, Daviaud E, Lund C (2019). Mental health system costs, resources and constraints in South Africa: a national survey. Health Policy Plan.

[21] Priebe S, Omer S, Giacco D, Slade M. Resource-oriented therapeutic models in psychiatry: Conceptual review. British Journal of Psychiatry vol. 204 Preprint at 10.1192/bjp.bp.113.135038 (2014). 10.1192/bjp.bp.113.135038 24692752

[22] van Loggerenberg F (2021). Feasibility, experiences and outcomes of using DIALOG+ in primary care to improve quality of life and mental distress of patients with chronic conditions: an exploratory non-controlled trial in Bosnia and Herzegovina, Colombia and Uganda. Pilot Feasibility Stud.

[23] Omer S, Golden E, Priebe S. Exploring the mechanisms of a patient-centred Assessment with a Solution focused Approach (DIALOG+) in the Community treatment of patients with psychosis: a process evaluation within a cluster-randomised controlled trial. (2016) 10.1371/journal.pone.0148415. 10.1371/journal.pone.0148415 PMC4747516 26859388

[24] Partners - NIHR Global Health Research Group. https://www.qmul.ac.uk/nihr-ghrg/partners/.

[25] Dialog+ - Resources. https://dialog.elft.nhs.uk/Resources.

[26] Priebe S (2019). Resource-oriented interventions for patients with severe mental illnesses in low-and middle-income countries: trials in Bosnia-Herzegovina, Colombia and Uganda. BMC Psychiatry.

[27] Gunn JM (2012). The association between chronic Illness, multimorbidity and depressive symptoms in an Australian primary care cohort. Soc Psychiatry Psychiatr Epidemiol.

[28] Le Cam L (1986). The Central Limit Theorem around 1935. Stat Sci.

[29] Jeste DV (2007). A new brief instrument for assessing decisional capacity for clinical research. Arch Gen Psychiatry.

[30] Dobson C. Conducting research with people not having the capacity to consent to their participation: A practical guide for researchers. (2008).

[31] Priebe S, Huxley P, Knight S, Evans S (1999). Application and results of the Manchester Short Assessment of Quality of Life (MANSA). Int J Soc Psychiatry.

[32] Shin C, Lee S-H, Han K-M, Yoon H-K, Han C (2019). Comparison of the usefulness of the PHQ-8 and PHQ-9 for screening for major depressive disorder: analysis of Psychiatric Outpatient Data. Psychiatry Investig.

[33] Löwe B et al. Validation and Standardization of the Generalized Anxiety Disorder Screener (GAD-7) in the General Population. vol. 46 (2008). 10.1097/MLR.0b013e318160d093 18388841

[34] Priebe S, Watzke S, Hansson L, Burns T (2008). Objective social outcomes index (SIX): a method to summarise objective indicators of social outcomes in mental health care. Acta Psychiatr Scand.

[35] Slatina Murga S (2021). Effectiveness of a structured intervention to make routine clinical meetings therapeutically effective (DIALOG+) for patients with depressive and anxiety disorders in Bosnia and Herzegovina: a cluster randomised controlled trial. Psychiatry Res Commun.

[36] Birabwa-Oketcho H et al. The effectiveness of a solution-focused approach (DIALOG+) for patients with severe mental Illness and Epilepsy in Uganda: a randomised controlled trial. Psychiatry Res Commun 3, (2023). 10.1016/j.psycom.2022.100097 PMC9995275 36911535

[37] García-Campayo J et al. Cultural adaptation into Spanish of the generalized anxiety disorder-7 (GAD-7) scale as a screening tool. Health Qual Life Outcomes 8, (2010). 10.1186/1477-7525-8-8 PMC2831043 20089179

[38] Šljivo A, Kulenović AD, Fear. Anxiety and depression among Bosnia and Herzegovina Citizens during the Third Wave of COVID-19. Iran J Psychiatry 18, (2023). 10.18502/ijps.v18i1.11407 PMC10163905 37159641

[39] Nakku JEM et al. Validity and diagnostic accuracy of the Luganda version of the 9-item and 2-item Patient Health Questionnaire for detecting major depressive disorder in rural Uganda. Global Mental Health 3, (2016). 10.1017/gmh.2016.14 PMC5314749 28596888

[40] Ssewanyana Y et al. Quality of life of adult individuals with intestinal stomas in Uganda: a cross sectional study. Afr Health Sci 21, (2021). 10.4314/ahs.v21i1.53 PMC8356576 34394325

[41] Vancampfort D et al. Mental contrasting and implementation of physical activity intentions in Ugandan primary care patients with mental health problems: a real-world intervention involving support partners. Psychiatry Res 307, (2022). 10.1016/j.psychres.2021.114335 34920396

[42] Kroenke K, Spitzer RL, Williams JB. W. The PHQ-9: validity of a brief depression severity measure. J Gen Intern Med 16, (2001). 10.1046/j.1525-1497.2001.016009606.x PMC1495268 11556941

[43] Wu Y et al. Equivalency of the diagnostic accuracy of the PHQ-8 and PHQ-9: a systematic review and individual participant data meta-analysis – ERRATUM. Psychol Med 50, (2020). 10.1017/S0033291719001314 PMC6954991 31298180

[44] Ali GC, Ryan G, De Silva MJ. Validated screening tools for common mental disorders in low and middle income countries: A systematic review. PLoS ONE vol. 11 Preprint at 10.1371/journal.pone.0156939 (2016). 10.1371/journal.pone.0156939 PMC4911088 27310297

[45] Cassiani-Miranda CA et al. Validity of the Patient Health Questionnaire-9 (PHQ-9) for depression screening in adult primary care users in Bucaramanga, Colombia. Rev Colomb Psiquiatr 50, (2021). 10.1016/j.rcp.2019.09.001 33648690

[46] ZZJZ I(Law on Healthcare.) Federation of Bosnia and Hertzegovina). https://www.zzjzfbih.ba/.

[47] Ministry of Health of the. Federation of Bosnia and Herzegovina. http://www.fmoh.gov.ba/.

[48] The Public Institution. Health Centre of Sarajevo Canton. http://judzks.ba/.

[49] POLÍTICA DE ATENCIÓN INTEGRAL EN SALUD Ministerio de Salud y Protección Social. Repositorio Institucional Digital Minsalud (RID) https://www.minsalud.gov.co/sites/rid/Lists/BibliotecaDigital/RIDE/DE/modelo-pais-2016.pdf (2016).

[50] Health M. of. Uganda health system assessment 2011. (2012).

[51] Mukasa N (2012). Uganda Healthcare system profile: background, organization, policies and challenges. J Sustainable Reg Health Syst.

[52] Leon AC, Davis LL, Kraemer HC (2011). The role and interpretation of pilot studies in clinical research. J Psychiatr Res.

[53] Demarzo MMP (2015). The efficacy of mindfulness-based interventions in primary care: a Meta-Analytic Review. The Annals of Family Medicine.

[54] Schmidt K (2016). Effect of a primary Care Management intervention on Mental Health–Related Quality of Life among survivors of Sepsis: a Randomized Clinical Trial. JAMA.

[55] Sarwer DB (2013). The impact of a primary care-based weight loss intervention on the quality of life. Int J Obes.

[56] Weinberger M (1995). A nurse-coordinated intervention for primary care patients with non-insulin-dependent Diabetes Mellitus: impact on glycemic control and health-related quality of life. J Gen Intern Med.

[57] Rosemann T (2005). Rationale, design and conduct of a comprehensive evaluation of a primary care based intervention to improve the quality of life of osteoarthritis patients. The PraxArt-project: a cluster randomized controlled trial [ISRCTN87252339]. BMC Public Health.

[58] Niesink A (2007). Systematic review of the effects of chronic Disease management on quality-of-life in people with Chronic Obstructive Pulmonary Disease. Respir Med.

[59] Mercer SW (2016). The CARE Plus study – a whole-system intervention to improve quality of life of primary care patients with multimorbidity in areas of high socioeconomic deprivation: exploratory cluster randomised controlled trial and cost-utility analysis. BMC Med.

[60] Lee W-J (2021). Effects of incorporating multidomain interventions into integrated primary care on quality of life: a randomised controlled trial. Lancet Healthy Longev.

[61] Smith SM, Soubhi H, Fortin M, Hudon C, O’Dowd T. Managing patients with multimorbidity: systematic review of interventions in primary care and community settings. *BMJ* 345, (2012). 10.1136/bmj.e5205 PMC3432635 22945950

[62] Watson LC (2013). Practice-based interventions addressing Concomitant Depression and Chronic Medical conditions in the primary care setting: a systematic review and Meta-analysis. J Prim Care Community Health.

